# Oil price uncertainty, oil pricing reform, and corporate profitability: The case of China

**DOI:** 10.1371/journal.pone.0297554

**Published:** 2024-02-02

**Authors:** Giang Thi Huong Vuong, Manh Huu Nguyen, Khanh Hoang

**Affiliations:** 1 Faculty of Finance, Ho Chi Minh University of Banking, Ho Chi Minh City, Vietnam; 2 Faculty of Accounting and Finance, Nha Trang University, Nha Trang City, Khanh Hoa Province, Vietnam; 3 College of Technology and Design, University of Economics Ho Chi Minh City, Ho Chi Minh City, Viet Nam; University of Kotli Azad Jammu and Kashmir Faculty of Management Sciences, PAKISTAN

## Abstract

This study investigates the impact of oil price uncertainty (OPU) on corporate profitability in China, the world’s largest crude oil consumer. Most importantly, we examine how the Chinese government’s oil price reform affects this relationship. Using the yearly data of Chinese-listed companies, we find that the uncertainty of oil prices negatively affects corporate profitability but positively impacts operating expenses from 2007 to 2020. This finding holds after robust tests, including alternative profitability metrics and endogeneity model. Most interestingly, implementing the 2013 market-oriented oil pricing reform amplifies the adverse impact of OPU on corporate profitability owing to increased operating costs in the post-2013 period. Moreover, the detrimental effect of uncertain oil prices on corporate profitability is less prominent for large-capitalized companies. This research adds to the body of knowledge on the factors affecting corporate profitability by highlighting the volatility effect of oil prices and government pricing mechanisms. The results offer grounds for legislators and corporate managers to consider how to control the uncertainty surrounding oil price matters to ensure stable corporate profitability.

## 1. Introduction

China’s central government has loosened price controls five times since 1998 in response to the country’s growing oil imports and consumption. This has increased China’s oil price’s responsiveness to global oil price fluctuations, particularly after this country implemented a market-oriented reform on March 27^th^, 2013. China has executed a new oil pricing mechanism in an effort to improve the efficiency and competitiveness of its domestic oil industry. Before the 2013 reform, the Chinese government controlled the price of oil, which was insufficiently representative of the overall condition of the market. Consequently, domestic oil producers often struggled to compete with imported oil, which was more synchronized with market movements. The 2013 oil pricing mechanism, which eliminated the 4% worldwide oil price fluctuation trigger point and shortened the price adjustment cycle from 22 to 10 working days, was a significant step toward market-oriented pricing for refined oil. The price of crude oil on the international market, domestic production costs, and China’s oil demand are the key factors determining the price of oil in China under the 2013 reform phase [1]. The 2013 reform helped improve the informativeness of oil prices in China and reduce the financial burden on the government [2], which previously had to subsidize the cost of domestic oil production.

The 2013 oil pricing mechanism, according to the National Development and Reform Commission (NDRC), is more adaptable to changes in the global oil market and allows the nation to make more effective utilization of its foreign resources to secure domestic oil supply [3]. [1] find that the oil price volatility of China is more tightly linked to the price volatility of global oil, and the trajectory of both is similar compared to before the market-oriented reform. An increase in oil prices directly affects commodity prices and inflation, leading to a reduction in consumption [4]. A decrease in demand could drop corporate profitability [5], resulting in less corporate investment [6]) or even boosting bankruptcy risk [7]. In addition, China is the largest oil importer in the world. The volatility impact of oil prices is likely to seriously affect the country’s trade balance and budget deficit [4]. Exacerbating oil prices leads to an increase in China’s import bill and a corresponding decrease in its foreign exchange reserves [8], which puts pressure on the country’s exchange rate and might lead to economic instability. In other words, oil price volatility amplifies macro risks.

**Fig 1:**
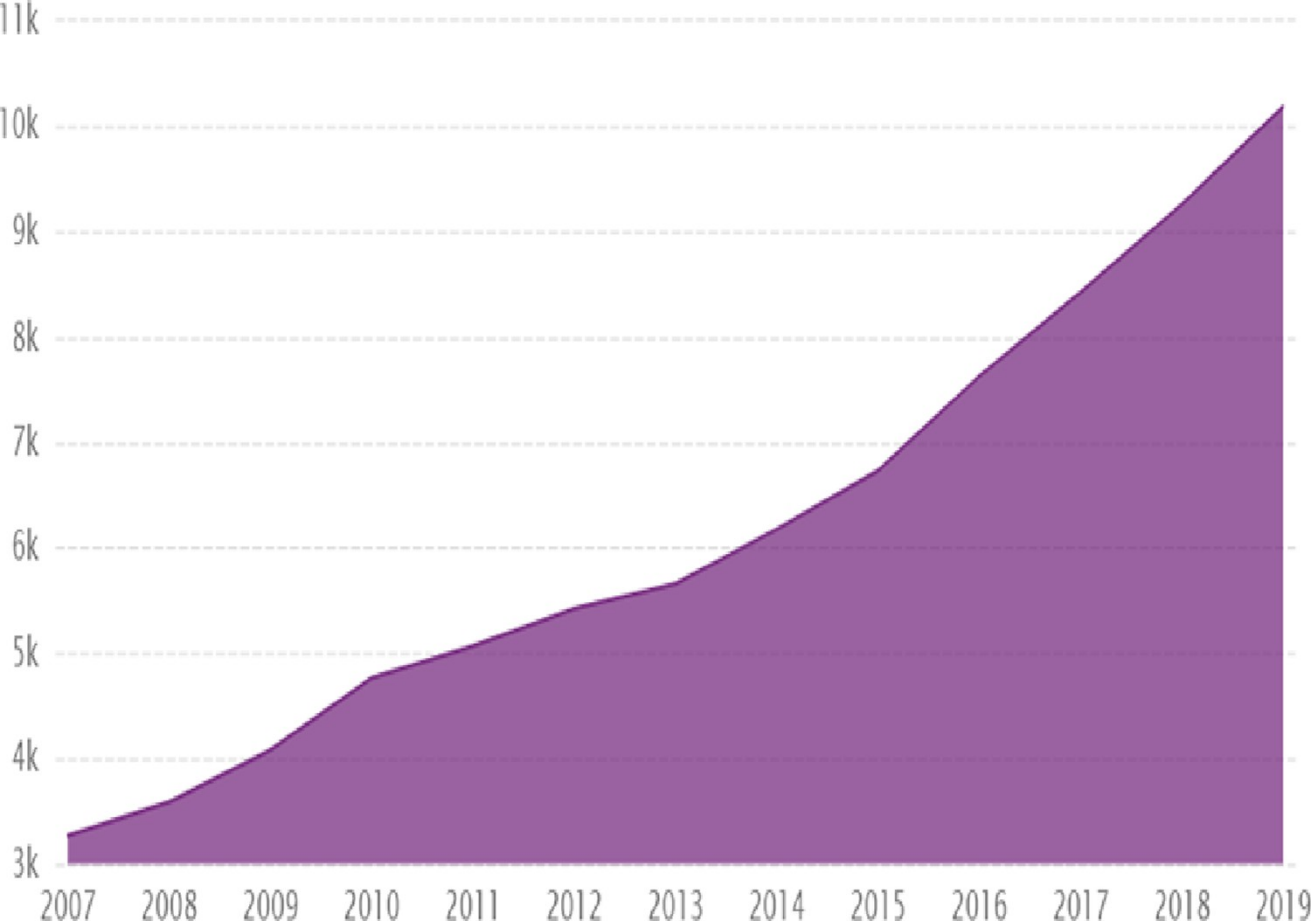
China’s crude oil import production (barrels/day) from 2007 to 2019. Source: U.S Energy Information Administration (EIA).

Fig 1 illustrates that China’s imported crude oil production increased continuously from 2007 to 2019, from over 3.2 million barrels per day to approximately 10.2 million barrels per day. As a result of its high oil consumption, China’s economy is increasingly vulnerable to fluctuations in oil prices on the world market. As the Chinese economy is heavily dependent on oil and fossil fuels, the production and financial resources of Chinese firms are sensitive to crude oil price uncertainty [9].

Existing literature has paid more attention to the themes surrounding the link between uncertainty and corporate outcomes. [10] argues that uncertainty factors cause higher expenses for firms, thereby decreasing profitability. Uncertainty might come from either firm-specific volatility or macroeconomic factor volatility. Firm-specific uncertainty consists of earnings volatility, cash flow volatility, and stock return volatility [11]. Macroeconomic volatility is considered political uncertainty [12], policy uncertainty [13, 14], and oil price uncertainty [5, 15]. [16] state that the price of crude oil is a key factor in consumer price variations. The negative impact of oil price shocks on consumer prices is more pronounced in South African countries [17]. From a macro perspective, [6] explain how the uncertain impact of oil prices on consumer prices occurs through two channels: "supply-demand." This means that the volatility of crude oil prices is likely to affect enterprises’ input and output prices. In particular, this energy price uncertainty might lead firms to postpone investments [2, 6], hold more cash [18], alleviate debts in the capital structure [7], and reduce stock returns [19].

Many studies have examined the uncertain effect of oil prices on corporate decisions [7, 9, 20, 21]; macroeconomic factors [22–24]; and the equity market [25, 26]. The research on the relationship between unpredictable oil prices and corporate profitability in emerging markets is more interesting because macroeconomic volatility in these markets tends to be higher than in developed markets [10]. Prior studies have shown that crude oil price volatility is negatively related to corporate profits in the US market [5] and among GCC-listed firms [15]. China’s country standing out by a state dominance and an administrative monopoly over the petrochemical sector is an experimental objective with intriguing and promising results on the oil price uncertainty-profitability link.

In recent research, [27] examine the moderating effect of corporate governance on the link between oil price variability and Chinese firms’ performance. They clarify how to differentiate this nexus between energy-related and non-energy-related sectors. Still, their paper has three limitations, as follows. Firstly, [27] have not indicated the specified channel that makes firm profits lower when the change in oil prices surges. Secondly, “China changed its oil pricing mechanism in March 2013. It may adjust domestic oil prices every 10 working days regardless of how much international oil prices change, unless price changes in international oil markets are not more than 50 yuan per ton”. (Page. 263, [1]). The role of the 2013 Chinese oil pricing reform in the relationship between oil price volatility and firm performance has not yet been shown. Thirdly, the adverse impact of oil price fluctuations on corporate profitability, which varies across the firm’s market value levels, is unexplored. By investigating the interrelationship between uncertainty and cost-profitability, the roles of the 2013 oil pricing reform, and the firm’s market cap, our study provides a multidimensional insight into the effect of uncertain oil prices on the Chinese listed companies’ profitability. It enriches the existing literature and offers valuable insights for corporate stakeholders and policymakers in China.

Our study looks into the connection between changes in oil prices and company profitability. Additionally, based on the "demand-supply" effect and the effect of oil shocks on input and output prices, we discover how oil price instability affects the operational expenses of Chinese companies. Thirdly, to take into account the significant variation of the 2013 oil pricing mechanisms for Chinese companies, we analyze the changes in the effect of unstable oil prices on the profitability of Chinese firms in the pre- and post-2013 oil-pricing reform periods. Finally, we implement a further analysis to determine the controlling role of the company’s market capitalization in the nexus between uncertain oil prices and corporate profitability.

We employed multiple fixed effect estimators and a range of robustness tests for a panel sample of Chinese listed firms covering the period 2007–2020. Empirical results document the detrimental impact of unpredictable oil prices on corporate profitability. Specifically, one standard deviation increase in OPU is associated with a 0.160–0.281 standard deviation decrease in the return-on-assets ratio, implying that the effect is substantial. When we regress unsure oil prices against the cost-income ratio, we find that one standard deviation increase in OPU is associated with a 0.054–0.096 standard deviation increase in the return-on-assets ratio. These findings support our conjecture that oil price instability deteriorates the corporate profitability of Chinese firms by inflating operating costs. Our primary results hold after a variety of corporate profitability measurements, model specifications, and the instrumental variable approach. Additional tests indicate that a negative effect of unsure oil prices on corporate profitability is more manifest after the 2013 oil pricing reform, while this effect is significantly more positive pre-reform. This finding indicates a challenge that Chinese firms must encounter when the government enacts new regulations adjusting domestic oil prices. Moreover, the firm’s market value plays a moderating role in the relationship between the instability of oil prices and corporate profitability in China. High-cap firms are less vulnerable to the negative effects of oil price uncertainty on their corporate profitability.

This study contributes to the current literature surrounding corporate profitability and oil price variations in three aspects. Firstly, our findings provide a new insight into how oil price uncertainty impairs corporate profitability in the context of China, a communist country with strong government control over oil and energy prices. And the positive correlation between operational costs and increasing oil price volatility is a major cause. Secondly, our study opens a new insight into the 2013 oil pricing reform in China. It is not denied that this reform left the Chinese domestic oil market closely linked to changes in the global oil markets [1]. We illustrate the obstacles faced by Chinese enterprises and the substantial effect that the volatility of oil prices has had on their profitability since the enactment of a new oil pricing mechanism by comparing the pre-reform and post-reform periods of oil pricing in China. To the best of our knowledge, this is the first study to indicate this phenomenon since the reform. Ultimately, large caps might tranquilize the adverse effects of unpredictable oil prices on Chinese company profitability. This finding discloses that listed firms with large caps are more favorable to issuing equity to cope with the detrimental influence of unstable oil prices.

The rest of this paper proceeds as follows: Section 2 reviews the literature and develops hypotheses. Section 3 discusses variable measurements, empirical models, and data. Section 4 reports the empirical results. Section 5 concludes the paper.

## 2. Literature review and hypothesis development

Corporate profitability is a key concern for any business and its managers, as both the company and its managers seek to maximize profits to achieve long-term, sustainable development goals [9]. Researchers have frequently studied the changes in a company’s profit patterns over time, as well as the influence of exogenous and endogenous factors on corporate profitability. Many studies have focused on firm-specific factors [28–30], corporate governance [27], industry-specific factors [31, 32], and macroeconomic factors [33, 34] on corporate profitability. The effect of macroeconomic factors like inflation, exchange rates, and economic growth on profitability has been extensively researched in traditional studies, although the results have varied depending on the samples used.

From a macro-uncertainty perspective, some research has looked at how macroeconomic uncertainty affects business profitability and economic efficiency, especially in developed countries. [35] find that exchange rate volatility has a negative impact on firm profitability in US multinational firms; conversely, [36] discover that exchange rate uncertainty has an unclear impact on profit growth levels. In an emerging market. [10] reveal that increased macroeconomic uncertainty negatively impacts the profitability of manufacturing firms. Recent studies concentrate on the relationship between economic policy uncertainty (EPU) and firm profitability in both emerging and developed markets. For example, [37] suggest that when EPU increases, corporate investments decrease, leading to lower profits and the performance of Chinese firms. [38] realize that an increase in policy uncertainty leads to an increase in investors’ risk sensitivity, causing a decrease in the performance of US firms. There is substantial proof that the uncertainty of economic policy lowers European enterprises’ performance [39]. Firm values have been pointed to fall amid surged climate policy uncertainty and geopolitical risks [14, 40].

In general, empirical evidence documents that macroeconomic fluctuations negatively affect corporate profitability in most markets around the world. The growing body of literature on oil price shocks motivates us to explore the link between oil price volatility and Chinese companies’ profitability. Moreover, the close connection between the domestic oil market and the international oil market has become tighter since the oil price reform on March 27^th^, 2013. Based on the above conjectures, we pose the following hypothesis:

**Hypothesis 1:** Corporate profitability is negatively associated with oil price uncertainty

The majority of previous studies have concentrated on the unstable effects of oil prices on investment decisions [6]; corporate capital structure [7]; corporate risk-taking [41]; corporate cash holdings [18]; or individual stock returns [1, 42]. All their findings confirm the adverse impact of oil price fluctuations in both emerging and developed markets. Overall, the evidence suggests that oil price instability creates unfavorable conditions for an enterprise’s activities. The literature on the effect of unstable oil prices on corporate profitability is comparatively scarce. To our best knowledge, the negative impact of crude oil price changes has been shown on US firm performance [5] and GCC firm performance [15]. From an expenditure perspective, [10] argues that external shocks from macroeconomic factors could lead to an increase in operating costs, which might directly decrease profits at the firm level. As uncertainty in oil prices increases, the fixed cost of production also increases [43], resulting in an overall increase in business operating costs. Both predicted and unanticipated fluctuations in China’s oil prices had a significant impact on the general commodities market [44]. China has become the world’s biggest oil importer, surpassing the United States in 2013. According to the Energy Information Administration (EIA), China’s oil consumption surpassed production by 6.3 million barrels per day in 2013. This suggests that oil prices will likely account for a significant portion of China’s economic spending. Drawing on the aforementioned arguments, we hypothesize that changes in oil prices have a detrimental impact on company profitability in China by raising operating costs.

**Hypothesis 2:** Cost-income ratio is positively associated with oil price uncertainty

The instability of oil prices leads to uncertainty in expected production costs, which is important for firms to estimate their profits. The market determines oil prices, and changes in the underlying factors could lead to changes in production costs, operating costs, and expected sale prices for enterprises. Before 1998, China’s government strictly regulated refined oil prices in an effort to mitigate oil price volatility and minimize the negative impact of international oil prices on the country’s economy. Some studies have examined the unexpected effects of this strict control on China’s economic growth [3] and shocks in international oil prices [45]. Since 1998, refined oil prices in China have been market-oriented due to increasing domestic oil consumption and pressure to import oil. However, this pricing reform has also increased the dependence of Chinese refined oil prices on international oil prices. The relationship between China’s stock market and the international oil market has been consolidated since 2013. There has been a lot of literature focusing on the role of the 2013 petroleum pricing reform on the connection between oil price volatility and stock returns [18, 46], the relationship between the OVX index and stock performance [47], the link between uncertain oil prices and corporate investment [2], and the relationship between oil price variations and corporate leverage [7]. Our paper expands our understanding of the influence of the 2013 oil pricing reform on the relationship between unstable oil prices, corporate profitability, and the operational expenses of Chinese firms. To do these, two hypotheses, H3a and H3b, are developed.

**Hypothesis 3a:** A negative relationship between corporate profitability and oil price uncertainty is amplified by the 2013 oil pricing reform
**Hypothesis 3b:** A positive relationship between operating costs and oil price uncertainty is strengthened by the 2013 oil pricing reform

Since 2007, the Chinese firm’s market value has significantly increased [48, 49], showing the crucial role of market equity financing. As reported by [50], there is a positive correlation between the volatility of international oil prices and the volatility of the Chinese stock market spanning 2003 to 2020. In other words, the rising volatility of oil prices is closely related to the turbulence in the stock market. Large-cap firms have an easier time obtaining outside funding sources and overcoming past obstacles when macroeconomic conditions are disastrous [51, 52]; that is one benefit of the market-capitalized scale. Listed firms regularly comply with their financial needs by issuing more shares than debts [7] to minimize bankruptcy risk when retained earnings drop under pressure from the exceeding oil price volatility. As a result, these firms have stronger resilience to external shocks (macro-uncertainties) compared to their counterparts [18].

The shock to oil prices diminishes the profits of Chinese firms because of increased expenses for operations, as Hypothesis 1 is reviewed. The theory of Pecking order states that Chinese companies tend to use external financing in greater amounts if they lack internal capital [49, 52]. Chinese large-cap companies prioritize issuing shares in light of their scale advantages. In this way, they react to market shocks and overcome obstacles. The above arguments predict that the detrimental effect of oil price volatility on corporate profitability is less pronounced for high-cap companies. We build the fourth hypothesis to predict the mediating role of market value in the link between oil price instability and Chinese firms’ profitability, as follows:

**Hypothesis 4:** The detrimental effect of oil price volatility on corporate profitability is less apparent for firms with large caps

## 3. Data, measurement of variables, and experimental models

### 3.1. Data

In 2005 and 2006, many listed firms in China converted from non-tradable to tradable in a restructuring of the Chinese stock market. Consequently, our panel sample for this study was collected starting in 2007, due to the significant changes in China’s stock market structure [53]. Our paper investigates the impact of unpredictable oil prices on corporate profitability using a panel sample of listed firms on the Shanghai and Shenzhen Stock Exchanges from 2007 to 2020. We exclude financial and real estate companies according to the classification of the China Securities Regulatory Commission (CSRC). Our sample is limited to firms with total assets higher than zero and positive operating costs, and we also exclude firms with a book leverage ratio above one to minimize anomalies in their capital structure. After completing these screening processes, our final sample consists of an unbalanced panel dataset of 9,826 firm-year observations for 768 firms. Annual financial reports were obtained from the Taiwan Economic Journal (TEJ) database.

Our objective is to investigate how crude oil price fluctuations affect the profitability of businesses. For crude oil West Texas Intermediate (WTI) futures, we use the daily closing prices from the Thomson Reuters Eikon (Datastream database) and the CBOE Crude Oil Bollinger Band® (COBE) volatility index. Plus, we use both of the EPU indices for the US and Russia introduced by [54] as well as the Chinese Economic Policy Uncertainty indicator created by [55] to deal with the endogeneity issue in the baseline model. The website (http://www.policyuncertainty.com) provided these EPU indicators.

### 3.2. Measurement of variables

#### 3.2.1. Corporate profitability

The main objective of our study is to analyze the effect of oil price volatility on corporate profitability from an operational cost standpoint. According to previous research, return on total assets (ROA) is a common measure of corporate profitability [29, 34, 56, 57]. To enhance the robustness of our results, we also use two additional measures of corporate profitability: return on equity (ROE) and return on sales (ROS) as alternatives to ROA in the baseline model. Another dependent variable in our paper is the cost-to-income ratio (CIR) which calculates the spending costs for generating each dollar of income. Estimated coefficient of CIR variable allows us to assess the uncertain impact of oil prices on the cost management efficiency of the firm and enlightens Hypothesis 2.

#### 3.2.2 Oil policy uncertainty

There are three methods for measuring oil price uncertainty (OPU): the standard deviation of oil price variations, the conditional variance from a GARCH model, and the implied volatility of crude oil prices. The first two methods are based on historical oil prices, which could be a disadvantage because they might not accurately capture information about future oil price volatility [47]. In contrast, the COBE crude oil implied volatility (OVX) index is a newer measure that takes into account both historical values and future information on oil prices [18, 58]. This index is considered superior to the other two measures and has gained attention among researchers [7, 18]. In our research, we use two measures of oil price volatility: the standard deviation of change in daily crude oil WTI futures prices (VOPSD) and the mean of the daily OXV index. We compare the impact of both measures on corporate profitability and cost management efficiency to clarify the robustness of main results. Following [59], annual volatility is defined as follows:

$${VOPSD}_{t}=\sqrt{\frac{1}{n-1}\sum_{i}^{n}{{(r}_{i,t}-\underline{r})}^{2}}*\sqrt{n}$$ Eq (1)

where *r*_*i*_ is the return of the closing price of the crude oil WTI futures on the day (i), *r* is the average of the total change in daily closing oil price for the year (t), and n is the number of trading days in the year (t).

According to [7], annual OPU is computed based on the daily COBE crude oil implied volatility index, as follows:

$${OVX}_{t}=\frac{1}{n}\sum_{i}^{n}{OVX}_{i,t}$$ Eq (2)

where *OVX*_*i*,*t*_ denotes the daily implied volatility index on the day (i) of the year (t),and n denotes the number of trading days in the year (t).

#### 3.2.3. Descriptive statistics

Table 1 presents summary statistics for the used variables. The definition of all variables is shown in Table 2. Corporate profitability is defined as the return on total assets (ROA). Net income, on average, accounts for 3.88% of their total assets. We also consider two additional measures of corporate profitability: the return on equity (ROE) and the return on sales (ROS). The mean values for ROE and ROS are 6.56% and 6.88%, respectively. To capture OPU, we use two proxies: the VIX options based OPU index (VOPSD) and the CBOE crude oil volatility index (OVX). The average value for VOPSD is 4.6526 with a standard deviation of 6.5770, while the average value for OVX is 38.8792 with a standard deviation of 11.4432. In Table 3, we present the correlation coefficients between each pair of variables. We do not find any significant multi-collinearity among the variables. The correlation coefficient between OPU (as measured by either VOPSD or OVX) and ROA, as well as the profitability variables (ROE and ROS), is negative. This suggests that an increase in OPU is associated with a decrease in corporate profitability. On the other hand, the correlations between OPU and the cost-income ratio (CIR) are positive, indicating that an increase in OPU is associated with a higher proportion of input costs relative to output costs for the firm.

**Table 1:** Summary statistics.

Variable	Obs.	Mean	Std. Dev.	Min	Max
ROA	9,826	0.0389	0.0614	-0.8414	0.5901
ROE	9,826	0.0656	0.1292	-0.9980	0.9892
ROS	9,826	0.0688	0.1481	-0.9955	0.9551
CIR	9,826	0.7398	0.1755	0.0003	16.494
LEV	9,826	0.1676	0.1454	0.0000	0.8056
SIZE	9,826	15.158	12.929	10.783	19.866
TANG	9,826	0.2539	0.1711	0.0000	0.9542
GROWTH	9,826	2.5845	2.2967	0.4429	28.958
LIQ	9,826	0.1752	0.1287	0.0001	0.9794
CF	9,826	0.0516	0.0787	-0.4963	0.9014
VOPSD	9,826	4.6483	6.5709	1.4688	27.989
OVX	9,826	3.6174	0.2936	3.1112	4.1743
EPU_CHINA	9,826	4.9535	0.5384	3.9209	5.9671

**Table 2:** Definitions of all variables.

Variable		Definitions	Formula
Corporate profitability	ROA	Return on Assets	Net income to total assets
	ROE	Return on Equity	Net income to total equity
	ROS	Return on Sales	Net income to total sales
Cost management efficiency	CIR	Cost Income Ratio	Operating costs to net sales
Control variables	LEV	Leverage	Total liability to total assets
	SIZE	Firm Size	Natural logarithm of total assets
	TANG	Asset Structure	Tangible assets to total assets
	GROWTH	Tobin’s Q ratio	Market capitalization plus total liability to book value of total assets
	LIQ	Liquid ratio	Cash and cash equivalent to total assets
	CF	Cash flow	Cash from operations to total assets
Oil price uncertainty	VOPSD	Oil price futures Volatility	The standard deviation of daily closing crude oil WTI futures price for the year (t)
	OVX	COBE implied oil price volatility	An average of daily OVX index for the year (t)
Economic policy uncertainty	EPU_CHINA	Chinese economic policy uncertainty	An average of the monthly Chinese EPU index during the year (t)
	EPU_US	United States economic policy uncertainty	An average of the monthly US EPU index during the year (t)
	EPU_RUS	Russian economic policy uncertainty	An average of the monthly Russian EPU index during the year (t)

**Table 3:** Pair correlations.

Variable	ROA	ROE	ROS	CIR	LEV	SIZE	TANG	GROWTH	LIQ	CF	VOPSD	OVX	EPU_CHINA
ROA	1.0000												
ROE	0.8669***	1.0000											
ROS	0.7677***	0.6751***	1.0000										
CIR	-0.4311***	-0.2986***	-0.5077***	1.0000									
LEV	-0.2892***	-0.1705***	-0.2127***	0.2917	1.0000								
SIZE	0.0265***	0.0951***	0.0505***	0.1124***	0.2400***	1.0000							
TANG	-0.0976***	-0.0933***	-0.0765***	0.1408***	0.3313***	0.0832***	1.0000						
GROWTH	0.1785***	0.0882***	0.1059***	-0.2846***	-0.2794***	-0.4751***	-0.1393***	1.0000					
LIQ	0.2576***	0.1624***	0.2042***	-0.1974***	-0.3402***	-0.1672***	-0.3557***	0.2103***	1.0000				
CF	0.3839***	0.3007***	0.2381***	-0.2318***	-0.1638***	0.0648***	0.2140***	0.0554***	0.1761***	1.0000			
VOPSD	-0.0498***	-0.0386***	-0.0447***	0.0104	-0.0327***	0.1337***	-0.0439***	-0.0627***	-0.0116	0.0227	1.0000		
OVX	-0.0099	-0.0074	-0.0084	0.0040	-0.0042	0.0079	-0.0096	0.0192	0.0483***	0.0406***	0.6333***	1.0000	
EPU_CHINA	-0.0766***	-0.0572***	-0.0509***	-0.0168*	-0.0761***	0.3106***	-0.1003***	-0.1965***	-0.0665***	0.0027	0.5065***	0.2586***	1.0000

Note: P-value is shown in parentheses.

**Fig 2:**
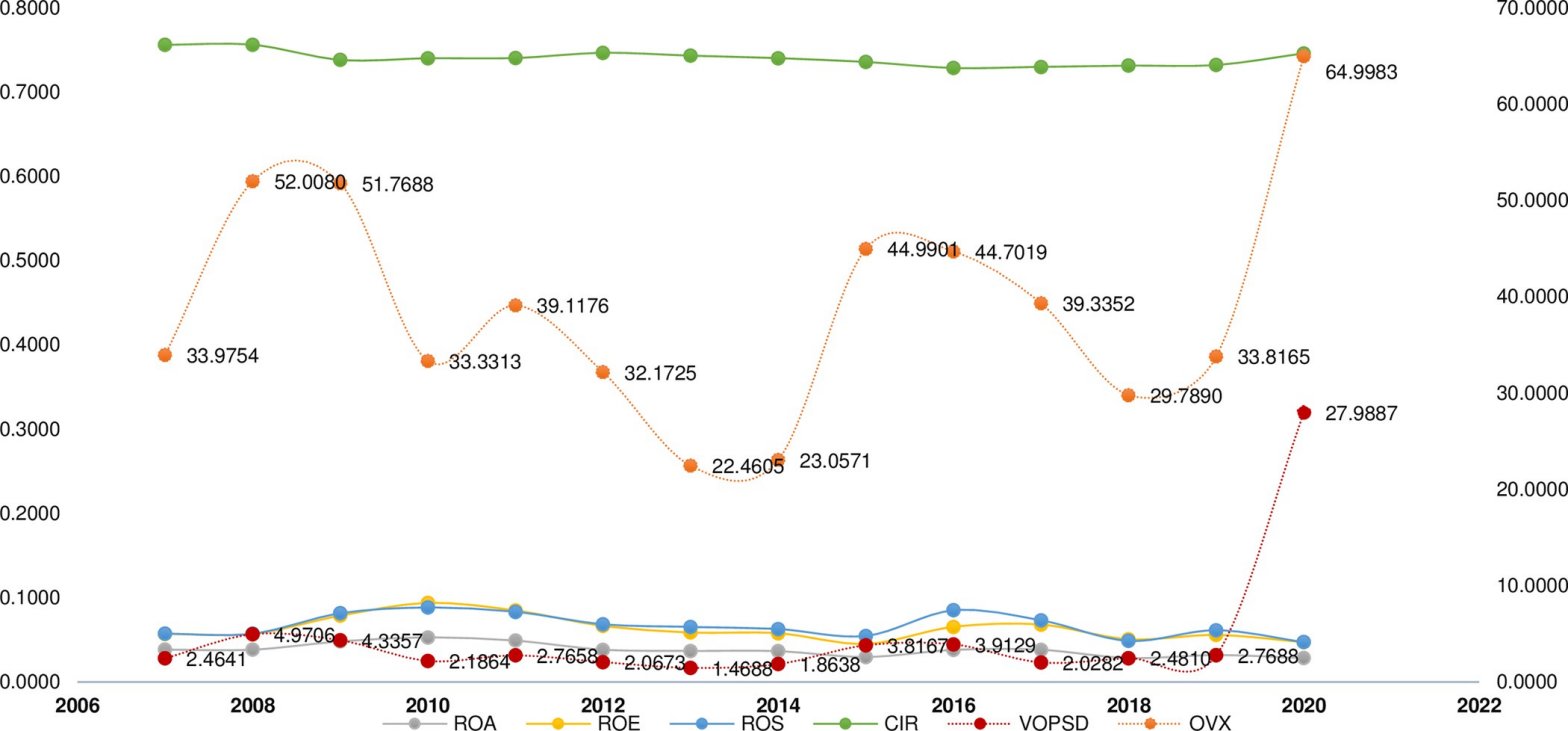
Oil price uncertainty, corporate profitability, and cost-income ratio of Chinese firms from 2007 to 2020. Note: Left-vertical axis displays the unit of corporate profitability and CIR variables while right-vertical axis exhibits the unit of oil price uncertainty variables.

Fig 2 shows the trends of OPU (OPU), profitability ratios, and the cost-income ratio (CIR) in Chinese firms over the period 2007–2020. From Fig 2, it is obvious that the three measures of OPU, VOPSD, and OVX have similar levels of volatility. On the other hand, the three corporate profitability variables—ROA, ROE, and ROS—display a homogeneous trend over time. While OPU tends to vary in a similar manner to the CIR over time, the profitability of Chinese enterprises appears to change inversely with OPU. In other words, as OPU increases, corporate profitability tends to decrease. In general, Fig 2 provides a preliminary understanding of the relationship between corporate profitability and OPU in Chinese firms.

### 3.3. Experiment models

To investigate the nexus between oil price uncertainty and corporate profitability, we estimate the baseline model as written below:

$${ROA}_{i,t}=\alpha +\beta_{1}{OPU}_{t}+\sum {CONTROL}_{i,t}+\lambda_{i}+\omega_{t}+\varepsilon_{i,t}$$ Eq (3)

where *ROA*_*i*,*t*_ denotes the return on assets of the firm (i) in the year (t), primarily representative of firm profitability. *OPU*_*t*_ mainly represents oil price volatility in the year (t). In this paper, *VOPSD*_*t*_ and *OVX*_*t*_ are two proxies of oil price volatility. The *β*_1_ coefficient shows the impact of OPU on corporate profitability. As stated in Hypothesis 1, we expect that the *β* value is negative and significant. ∑*CONTROL*_*i*,*t*_ is the vector of firm-level control variables. Following the corporate profitability literature, we control for the firm-specific variables that likely affect profitability. The control variables include financial leverage (LEV), firm size (SIZE), asset structure (TANG), growth opportunity (GROWTH), liquid ratio (LIQ), and cash flow (CF) [29, 60, 61]. Table 2 presents a description of the variables used in this study.

To examine Hypothesis 2, we replace the dependent variable in Eq (3) with the cost-income ratio (CIR) and then re-estimate the baseline model. In Hypothesis 2, we expect the estimated coefficients of the VOPSD and OVX variables to be positive and significant. To test Hypotheses 3a and 3b, we split the sample into pre- and post-2013 reforms, considering the oil pricing reform as a policy shock. We turn our attention to the impact of oil price instability on the profitability (ROA) and operating costs (CIR) of Chinese enterprises before and after the 2013 oil pricing mechanism. Hence, the change in signs of the two coefficients of the VOPSD and OVX variables in the two sub-periods is expected to shed light on Hypotheses 3a and 3b.

Finally, to test Hypothesis 4, we use the interaction variable between VOPSD and OVX with the HighCAP variable, as stated in the following model. Then, we evaluate the significance and sign of the coefficients of the interaction terms.

$${ROA}_{i,t}=\alpha +\beta_{1}{HighCAP}_{i,t}+\beta_{2}{HighCAP}_{i,t}*{OPU}_{t}+\beta_{3}{OPU}_{t}+\sum {CONTROL}_{i,t}+\lambda_{i}+\omega_{t}+\Omega_{i,t}$$ Eq (4)

where *HighCAP*_*i*,*t*_ variable denotes a listed company (i) with large caps or great market value in the year (t). The value of *HighCAP*_*i*,*t*_ variable equals 1 if the firm has a market capitalization above the mean of all market caps in our sample and 0 otherwise. The coefficient of the *HighCAP*_*i*,t_*OPU_*t*_ variable shows the moderating effect of the firm’s market value on the link between OPU and ROA variables. Given our expectation in Hypothesis 4, the *β*_2_ coefficient in Eq (4) is significantly positive.

In all specifications, we incorporate firm-fixed effects (*λ*_*i*_) because variations in corporate profitability are partially attributed to time-invariant but unobservable firm characteristics. Prior to fitting the slope coefficients, each company’s unique intercept is estimated. This allows for the control of unobservable, time-invariant sources of firm heterogeneity and focuses attention on the variance in corporate profitability over time at the individual firm level. To account for time-fixed effects that might have an impact on the profitability of the company, we incorporate time-fixed effects (*ω*_*t*_). *ε*_*i*,*t*_ and Ω_*i*,*t*_ are the error terms of empirical models. Additionally, standard errors used for significance assessment are corrected for heteroscedasticity and firm-level clustering because corporate profitability is expected to be associated with the enterprise over time.

## 4. Empirical analyses

### 4.1. Baseline analysis

Table 4 reports the estimated results for the influence of OPU on corporate profitability. Regression results from the baseline model are reported in two separate panels (Panels A and B) corresponding to two OPU variables (VOPSD and OVX). Estimating the baseline model using various techniques to confirm the robustness of the results. Column (1) represents the results from pooled OLS regression. We take into account both firm and time-fixed effects to eliminate the potential impact of the heterogeneity among companies and simultaneously check the effect of control variables over time in Column (2). Finally, we gather the standard errors at the firm-level and report the results in Column (3).

**Table 4:** Impact of Oil price uncertainty on corporate profitability (ROA).

Variable	Panel A: Oil price futures volatility (VOPSD)			Variable	Panel B: COBE implied oil price volatility (OVX)		
	(1)	(2)	(3)		(1)	(2)	(3)
VOPSD	-0.0006***	-0.0016***	-0.0016***	OVX	-0.0060***	-0.0610***	-0.0610***
	(0.0001)	(0.0001)	(0.0002)		(0.0017)	(0.0050)	(0.0087)
LEV	-0.0697***	-0.0946***	-0.0946***	LEV	-0.0675***	-0.0946***	-0.0946***
	(0.0050)	(0.0060)	(0.0092)		(0.0050)	(0.0060)	(0.0092)
SIZE	0.0052***	0.0132***	0.0132***	SIZE	0.0045***	0.0132***	0.0132***
	(0.0006)	(0.0011)	(0.0023)		(0.0006)	(0.0011)	(0.0023)
TANG	-0.0205***	-0.0425***	-0.0425***	TANG	-0.0187***	-0.0425***	-0.0425***
	(0.0047)	(0.0063)	(0.0107)		(0.0047)	(0.0063)	(0.0107)
GROWTH	0.0039***	0.0054***	0.0054***	GROWTH	0.0039***	0.0054***	0.0054***
	(0.0003)	(0.0003)	(0.0011)		(0.0003)	(0.0003)	(0.0011)
LIQ	0.0619***	0.0441***	0.0441***	LIQ	0.0635***	0.0441***	0.0441***
	(0.0053)	(0.0059)	(0.0083)		(0.0053)	(0.0059)	(0.0083)
CF	0.2045***	0.1547***	0.1547***	CF	0.2040***	0.1547***	0.1547***
	(0.0077)	(0.0078)	(0.0191)		(0.0077)	(0.0078)	(0.0191)
Constant	-0.0164***	-0.0491***	-0.0491**	Constant	0.0069	0.1621***	0.1621***
	(0.0058)	(0.0093)	(0.0192)		(0.0084)	(0.0178)	(0.0306)
Year effect	No	Yes	Yes	Year effect	No	Yes	Yes
Firm effect	No	Yes	Yes	Firm effect	No	Yes	Yes
Cluster	No	No	Yes	Cluster	No	No	Yes
R square	0.1180	0.1586	0.1586	R square	0.1147	0.1586	0.1586
Obs.	9,826	9,826	9,826	Obs.	9,826	9,826	9,826
Firms	768	768	768	Firms	768	768	768

Note: This table shows estimated results from the baseline model with ROA dependent variable. Pooled OLS regression results are reported in Column (1); Estimated results using both individual and time-fixed effects are shown in Column (2). Besides the estimated specification in Column (2), we clustered the errors by firms in Column (3). Standard errors are shown in parentheses in Columns (1) and (2). Robust standard errors are shown in parentheses in Column (3).

*, **, and *** denote significance at the 10%, 5%, and 1% levels, respectively.

It is proven that, at a 1% level, the instability of the oil price instability has a detrimental impact on the return on assets (ROA) of Chinese listed companies, according to the estimated findings of all specifications for the panel model in Table 4. As Hypothesis 1, the estimated results in both Panels A and B state that increased uncertainty in oil prices remarkably alleviates corporate profitability. Our results are robust to the findings of [5] and [15]. Negative LEV coefficients are consistent with the Pecking order theory (POT), suggesting that profitable firms use fewer debts in their capital structure [28, 29, 60]. Positive SIZE coefficients demonstrate that larger firms often create more profitability than small firms, which is in line with [28] findings. Negative TANG coefficients imply that firms spending more on tangible assets have lower profitability, which is in line with the findings of [60]. Positive GROWTH coefficients show that firms with high growth opportunities are more likely to earn more profits [12, 29]. Estimated LIQ coefficients are consistent with [34] study that profitable firms tend to have a high liquid ratio. The positive relation between CF and ROA variables is consistent with [62] research, implicating that profitable companies usually have abundant operating cash flows.

Furthermore, to prove that a rise in the uncertainty of oil prices certainly makes corporate profitability decline, we regress the baseline model by replacing the ROA-dependent variable with the ROE and ROS variables, respectively. Empirical results obtained by using two alternative variables of corporate profitability are reported in Table 5. In short, the estimated coefficients of unstable oil price variables in all columns of Table 5 strongly support the findings in Table 4. To put it differently, corporate profitability plummets when variations in oil prices tend to increase. Therefore, Hypothesis 1 is clarified.

**Table 5:** Impact of oil price uncertainty on alternative variables of corporate profitability (ROE and ROS).

Variable	Panel A: Oil price futures volatility (VOPSD)						Variable	Panel B: COBE implied oil price volatility (OVX)					
	ROE (1)	ROE (2)	ROE (3)	ROS (4)	ROS (5)	ROS (6)		ROE (1)	ROE (2)	ROE (3)	ROS (4)	ROS (5)	ROS (6)
VOPSD	-0.0013***	-0.0031***	-0.0031***	-0.0014***	-0.0037***	-0.0037***	OVX	-0.0118***	-0.1238***	-0.1238***	-0.0103**	-0.1475***	-0.1475***
	(0.0002)	(0.0003)	(0.0004)	(0.0002)	(0.0003)	(0.0005)		(0.0040)	(0.0115)	(0.0176)	(0.0041)	(0.0121)	(0.0190)
LEV	-0.0770***	-0.1364***	-0.1364***	-0.1279***	-0.1738***	-0.1738***	LEV	-0.0724***	-0.1364***	-0.1364***	-0.1228***	-0.1738***	-0.1738***
	(0.0108)	(0.0141)	(0.0251)	(0.0129)	(0.0148)	(0.0239)		(0.0108)	(0.0141)	(0.0251)	(0.0130)	(0.0148)	(0.0239)
SIZE	0.0136***	0.0235***	0.0235***	0.0138***	0.0338***	0.0338***	SIZE	0.0123***	0.0235***	0.0235***	0.0119***	0.0338***	0.0338***
	(0.0013)	(0.0026)	(0.0051)	(0.0016)	(0.0027)	(0.0047)		(0.0013)	(0.0026)	(0.0051)	(0.0016)	(0.0027)	(0.0047)
TANG	-0.0783***	-0.1221***	-0.1221***	-0.0535***	-0.1110***	-0.1110***	TANG	-0.0748***	-0.1221***	-0.1221***	-0.0489***	-0.1110***	-0.1110***
	(0.0099)	(0.0147)	(0.0227)	(0.0128)	(0.0154)	(0.0307)		(0.0099)	(0.0147)	(0.0227)	(0.0128)	(0.0154)	(0.0307)
GROWTH	0.0053***	0.0075***	0.0075***	0.0053***	0.0068***	0.0068***	GROWTH	0.0054***	0.0075***	0.0075***	0.0054***	0.0068***	0.0068***
	(0.0006)	(0.0008)	(0.0017)	(0.0007)	(0.0009)	(0.0023)		(0.0006)	(0.0008)	(0.0017)	(0.0007)	(0.0009)	(0.0023)
LIQ	0.0637***	0.0334**	0.0334**	0.1734***	0.1446***	0.1446***	LIQ	0.0670***	0.0334**	0.0334**	0.1761***	0.1446***	0.1446***
	(0.0117)	(0.0136)	(0.0163)	(0.0134)	(0.0143)	(0.0183)		(0.0118)	(0.0136)	(0.0163)	(0.0134)	(0.0143)	(0.0183)
CF	0.4094***	0.3198***	0.3198***	0.2199***	0.1795***	0.1795***	CF	0.4084***	0.3198***	0.3198***	0.2178***	0.1795***	0.1795***
	(0.0173)	(0.0182)	(0.0330)	(0.0187)	(0.0191)	(0.0322)		(0.0173)	(0.0182)	(0.0330)	(0.0188)	(0.0191)	(0.0322)
Constant	-0.0542***	-0.0646***	-0.0646	-0.0590***	-0.1483***	-0.1483***	Constant	-0.0095	0.3641***	0.3641***	-0.0147	0.3626***	0.3626***
	(0.0123)	(0.0216)	(0.0395)	(0.0158)	(0.0227)	(0.0407)		(0.0187)	(0.0415)	(0.0517)	(0.0214)	(0.0435)	(0.0636)
Firm effect	No	Yes	Yes	No	Yes	Yes	Firm effect	No	Yes	Yes	No	Yes	Yes
Year effect	No	Yes	Yes	No	Yes	Yes	Year effect	No	Yes	Yes	No	Yes	Yes
Cluster	No	No	Yes	No	No	Yes	Cluster	No	No	Yes	No	No	Yes
R square	0.0631	0.0957	0.0957	0.0665	0.0915	0.0915	R square	0.0601	0.0957	0.0957	0.0622	0.0915	0.0915
Obs.	9,826	9,826	9,826	9,826	9,826	9,826	Obs.	9,826	9,826	9,826	9,826	9,826	9,826
Firms	768	768	768	768	768	768	Firms	768	768	768	768	768	768

This table shows estimated results from the baseline model with ROE and ROS-dependent variables. Panels A and B reports empirical results with VOPSD and OVX variables, respectively. Estimated results from Pooled OLS regression are shown in Columns (1) and (4). Estimated results using both individual and time-fixed effects are shown in Columns (2) and (5). Besides, we clustered the errors by firms in Columns (3) and (6). Standard errors are shown in parentheses in Columns (1), (2), (4), and (5). Robust standard errors are shown in parentheses in Columns (3) and (6).

*, **, and *** denote significance at the 10%, 5%, and 1% levels, respectively.

According to the speculation of [10], when oil price instability tends to increase, firms are more likely to incur more costs, so their profits tend to decrease. In addition, oil price fluctuations affect consumer prices through “demand-supply” [6]. The cost-income ratio (CIR) is a measure of operational efficiency, usually used in the banking industry [63]. A rise in CIR indicates that operational expenses considerably boost revenue for companies. CIR is defined as the ratio of operating costs to total sales. To assert that the uncertainty of oil prices is positively related to corporate expenditures, we replace the ROA independent variable in the baseline model with the CIR dependent variable. Table 6 reports the estimated results for the influence of OPU variables on CIR variables. Both the VOPSD and OVX coefficients are positive and significant. In other words, the heightened uncertainty of oil prices makes a rise in operating costs outweigh the one in revenue, which results in significantly decreasing profitability for Chinese listed enterprises. These findings validate our second hypothesis. Unlike [27] study, our paper offers a convincing argument for why Chinese enterprises’ profitability tends to decline due to growing oil price uncertainty.

**Table 6:** Impact of oil policy uncertainty on cost-income ratio (CIR).

Variable	Panel A: Oil price futures volatility (VOPSD)			Variable	Panel B: COBE implied oil price volatility (OVX)		
	(1)	(2)	(3)		(1)	(2)	(3)
VOPSD	0.0007***	0.0015***	0.0015***	OVX	0.0060*	0.0582***	0.0582***
	(0.0001)	(0.0002)	(0.0004)		(0.0031)	(0.0090)	(0.0157)
LEV	0.1176***	0.1211***	0.1211***	LEV	0.1150***	0.1211***	0.1211***
	(0.0104)	(0.0110)	(0.0257)		(0.0104)	(0.0110)	(0.0257)
SIZE	-0.0145***	-0.0255***	-0.0255***	SIZE	-0.0133***	-0.0255***	-0.0255***
	(0.0014)	(0.0020)	(0.0056)		(0.0013)	(0.0020)	(0.0056)
TANG	0.0612***	0.0552***	0.0552*	TANG	0.0588***	0.0552***	0.0552*
	(0.0109)	(0.0114)	(0.0310)		(0.0109)	(0.0114)	(0.0310)
GROWTH	-0.0063***	-0.0068***	-0.0068***	GROWTH	-0.0063***	-0.0068***	-0.0068***
	(0.0006)	(0.0006)	(0.0019)		(0.0006)	(0.0006)	(0.0019)
LIQ	-0.0473***	-0.0371***	-0.0371*	LIQ	-0.0485***	-0.0371***	-0.0371*
	(0.0104)	(0.0106)	(0.0214)		(0.0104)	(0.0106)	(0.0214)
CF	-0.2110***	-0.1980***	-0.1980***	CF	-0.2096***	-0.1980***	-0.1980***
	(0.0142)	(0.0142)	(0.0263)		(0.0142)	(0.0142)	(0.0263)
Constant	0.8545***	0.9324***	0.9324***	Constant	0.8273***	0.7309***	0.7309***
	(0.0139)	(0.0169)	(0.0467)		(0.0175)	(0.0324)	(0.0540)
Firm effect	No	Yes	Yes	Firm effect	No	Yes	Yes
Year effect	No	Yes	Yes	Year effect	No	Yes	Yes
Cluster	No	No	Yes	Cluster	No	No	Yes
R square	0.0692	0.0748	0.0748	R square	0.0669	0.0748	0.0748
Obs.	9,826	9,826	9,826	Obs.	9,826	9,826	9,826
Firms	768	768	768	Firms	768	768	768

Note: This table reports empirical results from baseline model with CIR dependent variable. Pooled OLS regression results are reported in Column (1). Estimated results using both individual and time-fixed effects are shown in Column (2). Besides the estimated specification in Column (2), we clustered the errors by firms in Column (3). Standard errors are shown in parentheses in Columns (1) and (2). Robust standard errors are shown in parentheses in Columns (3).

*, **, and *** denote significance at the 10%, 5%, and 1% levels, respectively.

### 4.2. Further analysis

#### 4.2.1. The role of the 2013 oil pricing regime

In the pre-2013 period, the strict control of the Chinese government could mitigate the negative effect of uncertain oil prices on corporate profitability. However, the 2013 oil pricing reform might make Chinese firms suffer a massive fluctuation in costs, leading to an increase in the detrimental effect of unsure oil prices on corporate profitability. Following [7], the oil pricing reform was proposed on March 27^th^, 2013, promoting co-volatility between domestic and international oil prices. Thus, we split the whole period into two sub-periods. The former starts from 2007 to 2012 and is considered the pre-2013 reform. The latter spans from 2013 to 2020, referring to the post-reform period. Panels A and B of Table 7 represent empirical results of the effect of oil price uncertainty on corporate profitability, corresponding to two measures of unpredictable oil prices (VOPSD and OVX). Similarly, Panels A and B of Table 8 represent estimated results of the impact of oil price instability on corporate profitability, corresponding to two measures of unstable oil prices (VOPSD and OVX).

**Table 7:** Impact of oil price uncertainty on corporate profitability in the pre- and post-2013 periods.

**Panel A: Oil price futures volatility (VOPSD) as the explanatory variable**						
**Variable**	**The pre- 2013 reform period**			**The post- 2013 reform period**		
	**(1)**	**(2)**	**(3)**	**(1)**	**(2)**	**(3)**
VOPSD	0.0006	0.0477***	0.0477***	-0.0005***	-0.0008***	-0.0008***
	(0.0007)	(0.0077)	(0.0180)	(0.0001)	(0.0002)	(0.0001)
LEV	-0.0856***	-0.0993***	-0.0993***	-0.1020***	-0.1155***	-0.1155***
	(0.0074)	(0.0104)	(0.0166)	(0.0071)	(0.0149)	(0.0096)
SIZE	0.0132***	0.0210***	0.0210***	0.0092***	0.0076**	0.0076***
	(0.0011)	(0.0026)	(0.0043)	(0.0009)	(0.0036)	(0.0019)
TANG	-0.0290***	-0.0265**	-0.0265	-0.0260***	-0.0703***	-0.0703***
	(0.0073)	(0.0116)	(0.0179)	(0.0060)	(0.0163)	(0.0101)
GROWTH	0.0068***	0.0059***	0.0059**	0.0030***	0.0041***	0.0041***
	(0.0005)	(0.0007)	(0.0030)	(0.0004)	(0.0010)	(0.0005)
LIQ	0.0472***	0.0224**	0.0224	0.0350***	0.0408***	0.0408***
	(0.0078)	(0.0102)	(0.0145)	(0.0080)	(0.0129)	(0.0098)
CF	0.1351***	0.0886***	0.0886***	0.2362***	0.1727***	0.1727***
	(0.0108)	(0.0117)	(0.0211)	(0.0105)	(0.0274)	(0.0113)
Constant	-0.0693***	-0.2253***	-0.2253***	-0.0462***	-0.0089	-0.0089
	(0.0101)	(0.0315)	(0.0517)	(0.0083)	(0.0311)	(0.0163)
Firm effect	No	Yes	Yes	No	Yes	Yes
Year effect	No	Yes	Yes	No	Yes	Yes
Cluster	No	No	Yes	No	No	Yes
R square	0.0882	0.1051	0.1051	0.1058	0.1289	0.1289
Obs.	4,074	4,074	4,074	5,752	5,752	5,752
Firms	738	738	738	762	762	762
**Panel B: COBE implied oil price volatility (OVX) as the explanatory variable**						
OVX	0.0030	0.3475***	0.3475***	-0.0154***	-0.0201***	-0.0201***
	(0.0037)	(0.0559)	(0.1311)	(0.0020)	(0.0028)	(0.0039)
LEV	-0.0855***	-0.0993***	-0.0993***	-0.1046***	-0.1155***	-0.1155***
	(0.0073)	(0.0104)	(0.0166)	(0.0071)	(0.0096)	(0.0149)
SIZE	0.0132***	0.0210***	0.0210***	0.0104***	0.0076***	0.0076**
	(0.0011)	(0.0026)	(0.0043)	(0.0009)	(0.0019)	(0.0036)
TANG	-0.0290***	-0.0265**	-0.0265	-0.0265***	-0.0703***	-0.0703***
	(0.0073)	(0.0116)	(0.0179)	(0.0060)	(0.0101)	(0.0163)
GROWTH	0.0068***	0.0059***	0.0059**	0.0035***	0.0041***	0.0041***
	(0.0005)	(0.0007)	(0.0030)	(0.0004)	(0.0005)	(0.0010)
LIQ	0.0473***	0.0224**	0.0224	0.0342***	0.0408***	0.0408***
	(0.0078)	(0.0102)	(0.0145)	(0.0080)	(0.0098)	(0.0129)
CF	0.1354***	0.0886***	0.0886***	0.2339***	0.1727***	0.1727***
	(0.0108)	(0.0117)	(0.0211)	(0.0105)	(0.0113)	(0.0274)
Constant	-0.0779***	-1.3328***	-1.3328***	-0.0048	0.0524***	0.0524**
	(0.0179)	(0.2038)	(0.4536)	(0.0097)	(0.0155)	(0.0258)
Firm effect	No	Yes	Yes	No	Yes	Yes
Year effect	No	Yes	Yes	No	Yes	Yes
Cluster	No	No	Yes	No	No	Yes
R square	0.0882	0.1051	0.1051	0.1099	0.1051	0.1051
Obs.	4,074	4,074	4,074	4,074	4,074	4,074
Firms	738	738	738	738	738	738

Note: This table reports empirical results from the baseline model with ROA dependent variable in the pre-and post-2013 periods (2007–2012 and 2013–2020). VOPSD is a key independent variable. Pooled OLS regression results are reported in Column (1). Estimated results using both individual and time-fixed effects are shown in Column (2). Besides the estimated specification in Column (2), we clustered the errors by firms in Column (3). The standard error is shown in parentheses in Columns (1) and (2). Robust standard error is shown in parentheses in Columns (3). *, **, and *** denote significance at the 10%, 5%, and 1% levels, respectively.

Note: This table reports empirical results from the baseline model with ROA dependent variable in the pre-and post-2013 periods (2007–2012 and 2013–2020). OVX is a key independent variable. Pooled regression results are reported in Column (1). Estimated results using both individual and time-fixed effects are shown in Column (2). Besides the estimated specification in Column (2), we clustered the errors by firms in Column (3). The standard error is shown in parentheses in Columns (1) and (2). Robust standard error is shown in parentheses in Columns (3).

*, **, and *** denote significance at the 10%, 5%, and 1% levels, respectively.

**Table 8:** Impact of oil price uncertainty on operating costs (CIR) in the pre- and post-2013 periods.

**Panel A: Oil price futures volatility (VOPSD) as the explanatory variable**						
**Variable**	**The pre- 2013 reform period**			**The post- 2013 reform period**		
	**(1)**	**(2)**	**(3)**	**(1)**	**(2)**	**(3)**
VOPSD	-0.0010	-0.0598***	-0.0598**	0.0006***	0.0007***	0.0007***
	(0.0011)	(0.0112)	(0.0248)	(0.0001)	(0.0002)	(0.0003)
LEV	0.0438***	0.0165	0.0165	0.1477***	0.1326***	0.1326***
	(0.0145)	(0.0152)	(0.0285)	(0.0148)	(0.0156)	(0.0305)
SIZE	-0.0145***	-0.0327***	-0.0327***	-0.0088***	-0.0106***	-0.0106
	(0.0029)	(0.0038)	(0.0073)	(0.0023)	(0.0030)	(0.0085)
TANG	0.0136	-0.0138	-0.0138	0.1090***	0.1018***	0.1018**
	(0.0160)	(0.0169)	(0.0325)	(0.0147)	(0.0163)	(0.0410)
GROWTH	-0.0057***	-0.0043***	-0.0043	-0.0058***	-0.0055***	-0.0055***
	(0.0009)	(0.0010)	(0.0031)	(0.0007)	(0.0008)	(0.0017)
LIQ	-0.0670***	-0.0443***	-0.0443**	-0.0388**	-0.0446***	-0.0446*
	(0.0145)	(0.0148)	(0.0224)	(0.0155)	(0.0159)	(0.0268)
CF	-0.1188***	-0.1173***	-0.1173***	-0.2253***	-0.2085***	-0.2085***
	(0.0172)	(0.0170)	(0.0267)	(0.0182)	(0.0183)	(0.0344)
Constant	0.8819***	1.1614***	1.1614***	0.7904***	0.8075***	0.8075***
	(0.0260)	(0.0460)	(0.1001)	(0.0217)	(0.0265)	(0.0762)
Firm effect	No	Yes	Yes	No	Yes	Yes
Year effect	No	Yes	Yes	No	Yes	Yes
Cluster	No	No	Yes	No	No	Yes
R square	0.0342	0.0504	0.0504	0.0775	0.0790	0.0790
Obs.	4,074	4,074	4,074	5,752	5,752	5,752
Firms	738	738	738	762	762	762
**Panel B: COBE implied oil price volatility (OVX) as the explanatory variable**						
OVX	-0.0057	-0.4355***	-0.4355**	0.0129***	0.0185***	0.0185***
	(0.0057)	(0.0816)	(0.1807)	(0.0034)	(0.0045)	(0.0071)
LEV	0.0436***	0.0165	0.0165	0.1499***	0.1326***	0.1326***
	(0.0145)	(0.0152)	(0.0285)	(0.0148)	(0.0156)	(0.0305)
SIZE	-0.0143***	-0.0327***	-0.0327***	-0.0100***	-0.0106***	-0.0106
	(0.0028)	(0.0038)	(0.0073)	(0.0024)	(0.0030)	(0.0085)
TANG	0.0137	-0.0138	-0.0138	0.1080***	0.1018***	0.1018**
	(0.0160)	(0.0169)	(0.0325)	(0.0147)	(0.0163)	(0.0410)
GROWTH	-0.0056***	-0.0043***	-0.0043	-0.0064***	-0.0055***	-0.0055***
	(0.0008)	(0.0010)	(0.0031)	(0.0007)	(0.0008)	(0.0017)
LIQ	-0.0670***	-0.0443***	-0.0443**	-0.0379**	-0.0446***	-0.0446*
	(0.0145)	(0.0148)	(0.0224)	(0.0155)	(0.0159)	(0.0268)
CF	-0.1189***	-0.1173***	-0.1173***	-0.2219***	-0.2085***	-0.2085***
	(0.0172)	(0.0170)	(0.0267)	(0.0182)	(0.0183)	(0.0344)
Constant	0.8981***	2.5492***	2.5492***	0.7597***	0.7511***	0.7511***
	(0.0365)	(0.2975)	(0.6637)	(0.0219)	(0.0252)	(0.0660)
Firm effect	No	Yes	Yes	No	Yes	Yes
Year effect	No	Yes	Yes	No	Yes	Yes
Cluster	No	No	Yes	No	No	Yes
R square	0.0341	0.0504	0.0504	0.0754	0.0790	0.0790
Obs.	4,074	4,074	4,074	5,752	5,752	5,752
Firms	738	738	738	762	762	762

Note: This table reports empirical results from the baseline model with CIR dependent variable in the pre-and post-2013 periods (2007–2012 and 2013–2020). VOPSD is a key independent variable. Pooled OLS regression results are reported in Column (1). Estimated results using both individual and time-fixed effects are shown in Column (2). Besides the estimated specification in Column (2), we clustered the errors by firms in Column (3). The standard error is shown in parentheses in Columns (1) and (2). Robust standard error is shown in parentheses in Columns (3). *, **, and *** denote significance at the 10%, 5%, and 1% levels, respectively.

Note: This table reports empirical results from the baseline model with CIR dependent variable in the pre-and post-2013 periods (2007–2012 and 2013–2020). OVX is a key independent variable. Pooled OLS regression results are reported in Column (1). Estimated results using both individual and time-fixed effects are shown in Column (2). Besides the estimated specification in Column (2), we clustered the errors by firms in Column (3). The standard error is shown in parentheses in Columns (1) and (2). Robust standard error is shown in parentheses in Columns (3).

*, **, and *** denote significance at the 10%, 5%, and 1% levels, respectively.

Comparing OPU coefficients between the pre-reform period and post-reform period, we detect that before the promulgation of the 2013 oil pricing mechanism, the uncertainty of oil prices has a positive impact on profitability and a negative effect on the operating costs of Chinese firms. That implies the Chinese government’s control of oil prices contributes to easing the shocks of oil prices on the firm’s profits. After the 2013 oil pricing reform, oil price volatility has a negative effect on corporate profitability and a positive impact on the operating costs of Chinese firms. Our findings in Tables 7 and 8 prove that the adoption of market-oriented oil pricing has increased the adverse effect of oil price shocks on corporate profitability in China’s market. Therefore, Chinese corporate managers should carefully consider oil price uncertainty when planning profit maximization strategies.

Both [7, 9] reveal that the effect of oil price uncertainty on corporate debt has risen with the establishment of refined oil pricing market-oriented reform in 2013 due to the change in “demand-supply” in the commodity market. Their evidence implies that the Chinese have to diversify financing funds to deal with difficulties in this scenario. From a government perspective, research by [27] suggests that government interventions diminish the detrimental impact of oil price fluctuations on Chinese enterprise’s profitability. Through clarifying Hypotheses 3a and 3b, this research contributes to new evidence surrounding China’s 2013 oil pricing mechanism in relation to corporate profitability and operational costs.

#### 4.2.2. The moderating role of market capitalization scale

The enterprise’s market capitalization is mentioned as a regulatory factor in Hypothesis 4. Large-cap companies often favor issuing shares rather than debt when external financing costs rise due to erratic macroeconomic conditions and inadequate internal funding. In this session, we use an interaction variable between oil price uncertainty (VOPSD and OVX) and the HighCAP variable to investigate the controlling impact of market capitalization on the OPU-ROA nexus. Empirical results are reported in two panels of Table 9 regarding two measurements of oil price instability (VOPSD and OVX).

**Table 9:** Mediating role of market capitalization scale on the impact of oil price uncertainty on corporate profitability.

Variable	Panel A: Oil price futures volatility (VOPSD)			Variable	Panel B: COBE implied oil price volatility (OVX)		
	(1)	(2)	(3)		(1)	(2)	(3)
HighCAP	0.0203***	0.0177***	0.0177***	HighCAP	0.0741***	0.0442***	0.0442***
	(0.0020)	(0.0021)	(0.0024)		(0.0152)	(0.0152)	(0.0136)
HighCAP*VOPSD	0.0001	0.0004**	0.0004**	HighCAP*OVX	0.0146***	0.0078*	0.0078**
	(0.0002)	(0.0002)	(0.0002)		(0.0042)	(0.0041)	(0.0037)
VOPSD	-0.0006***	-0.0013***	-0.0013***	OVX	-0.0043**	-0.0548***	-0.0548***
	(0.0001)	(0.0001)	(0.0002)		(0.0020)	(0.0051)	(0.0090)
LEV	-0.0620***	-0.0881***	-0.0881***	LEV	-0.0597***	-0.0880***	-0.0880***
	(0.0050)	(0.0061)	(0.0091)		(0.0050)	(0.0061)	(0.0091)
SIZE	0.0003	0.0094***	0.0094***	SIZE	-0.0004	0.0092***	0.0092***
	(0.0007)	(0.0012)	(0.0025)		(0.0007)	(0.0012)	(0.0025)
TANG	-0.0202***	-0.0413***	-0.0413***	TANG	-0.0187***	-0.0418***	-0.0418***
	(0.0046)	(0.0063)	(0.0107)		(0.0046)	(0.0063)	(0.0107)
GROWTH	0.0026***	0.0047***	0.0047***	GROWTH	0.0027***	0.0046***	0.0046***
	(0.0003)	(0.0004)	(0.0011)		(0.0003)	(0.0004)	(0.0011)
LIQ	0.0617***	0.0450***	0.0450***	LIQ	0.0633***	0.0449***	0.0449***
	(0.0053)	(0.0058)	(0.0083)		(0.0053)	(0.0058)	(0.0082)
CF	0.2043***	0.1547***	0.1547***	CF	0.2037***	0.1547***	0.1547***
	(0.0076)	(0.0078)	(0.0192)		(0.0076)	(0.0078)	(0.0192)
Constant	0.0211***	-0.0238**	-0.0238	Constant	0.0385***	0.1677***	0.1677***
	(0.0067)	(0.0099)	(0.0209)		(0.0100)	(0.0185)	(0.0323)
Firm effect	No	Yes	Yes	Firm effect	No	Yes	Yes
Year effect	No	Yes	Yes	Year effect	No	Yes	Yes
Cluster	No	No	Yes	Cluster	No	No	Yes
R square	0.1248	0.1654	0.1654	R square	0.1230	0.1654	0.1654
Obs.	9,826	9,826	9,826	Obs.	9,826	9,826	9,826
Firms	768	768	768	Firms	768	768	768

Note: This table reports empirical results about the impact of oil price uncertainty on corporate profitability (ROA) across different firm’s market capitalizations. HighCAP is a dummy variable. If the firm’s market capitalization exceeds the average of all market capitalizations, the value of the HighCAP variable equals 1, in otherwise, equals 0. The moderating effect of a firm’s market capitalization on the nexus of OPU on the ROA variable is represented by the HighCAP*VOPSD and HighCAP*OVX interaction variables. Pooled OLS regression results are reported in Column (1). Estimated results using both individual and time-fixed effects are shown in Column (2). Besides the estimated specification in Column (2), we clustered the errors by firms in Column (3). The standard error is shown in parentheses in Columns (1) and (2). Robust standard error is shown in parentheses in Columns (3). *, **, and *** denote significance at the 10%, 5%, and 1% levels, respectively.

In both panels of Table 9, the HighCAP variable has a positive impact on the ROA variable, indicating that large-cap firms are more profitable than small-cap firms. The effect of the interaction variable between uncertain oil prices and HighCAP variables on the ROA variable is significantly positive. These results are consistent with Hypothesis 4 and provide supported evidence of the predictions of the Pecking order theory. To put it simply, the detrimental influence of unstable oil prices on corporate profitability is less obvious for firms with greater market value. Put another way, large-scale publicly traded companies prioritize issuing shares to overcome financing deficits when the effect of volatility in oil prices descends their profits.

#### 4.2.3. Two-stage least squares (2SLS) regression with instrumental variable (IV)

We address the endogeneity problem arising from omitted variable bias and enforce the main finding in the first and second hypotheses. In this paper, we use the average monthly US EPU and Russian EPU indexes as instrument variables in the two-stage least squares (2SLS) regression.

Previous studies have shown a significant relationship between the uncertainty of economic policy (EPU) and the instability of oil prices (OPU). [64, 65] indicate a positive connection between EPU and oil price shocks. In 2018, the US-China trade war resulted in strong fluctuations in crude oil prices. Wars and ongoing trade disputes had a significant impact on oil consumption during the unrest [66]. The high uncertainty dampened US consumer spending, which had a disproportionate impact on the business performance of Asian manufacturers, including China. In 2020, Russia was the largest supplier of oil to China, accounting for about 15.5% of China’s total oil imports. In 2021, it was China’s second-largest supplier. There were two major shocks in the international oil market in 2020 [67]. The COVID-19 pandemic caused oil demand to plummet as many countries were temporarily locked down. [67] find that higher uncertainty leads to greater oil price volatility due to the appearance of demand and supply shocks in both developed and emerging markets. Hence, OPU is strongly correlated with EPU in Russia and the US. However, there is a lack of evidence suggesting that either Russian EPU or US EPU has a direct impact on the profitability of Chinese-listed firms, implying the validity of our instrumental variables.

We report the empirical results of the 2SLS regression in Panels A and B of Table 10. We find that our instrument suffers from neither under-identification nor the weak-instrument problem in both panels. The second-stage regression results show that a negative link between unpredictable oil prices (VOPSD and OVX) and ROA variables remains significant (*P-value*<0.01); additionally, a positive nexus between uncertain oil prices (VOPSD and OVX) and CIR variables remains significant (*P-value*<0.01). The Anderson-Rubin Wald tests and the Stock-Wright LM S statistic corroborate the used instrument variables’ robustness. The potential endogeneity issues do not qualitatively affect our findings. On the whole, our main results are robust to different techniques.

**Table 10:** Two-stage least square (2SLS) regression with instrumental variable.

**Panel A: Endogeneity tests for the effect of VOPSD on ROA and CIR variables**				
**Variable**	**ROA is the dependent variable**		**CIR is the dependent variable**	
	**(1)**	**(2)**	**(1)**	**(2)**
*First stage*				
EPU_US	15.5467***		15.5467***	
	(0.0807)		(0.0807)	
EPU_RUS		9.3297***		9.3297***
		(0.0594)		(0.0594)
*Second stage*				
VOPSD	-0.0005***	-0.0017***	0.0007***	0.0017***
	(0.0004)	(0.0002)	(0.0002)	(0.0003)
LEV	-0.0683***	-0.0726***	0.1032***	0.1065***
	(0.0112)	(0.0113)	(0.0136)	(0.0137)
SIZE	0.0002	0.0027***	-0.0165***	-0.0184***
	(0.0009)	(0.0010)	(0.0019)	(0.0020)
TANG	-0.0344***	-0.0388***	0.0501***	0.0533***
	(0.0078)	(0.0080)	(0.0163)	(0.0162)
GROWTH	0.0040***	0.0039***	-0.0058***	-0.0057***
	(0.0008)	(0.0009)	(0.0011)	(0.0011)
LIQ	0.0612***	0.0614***	-0.0490***	-0.0491***
	(0.0071)	(0.0072)	(0.0128)	(0.0128)
CF	0.1548***	0.1595***	-0.2006***	-0.2040***
	(0.0150)	(0.0153)	(0.0201)	(0.0203)
Weak-instrument-robust inference:				
Anderson-Rubin Wald test *(P-value)*	0.0000	0.0000	0.0000	0.0000
Stock-Wright LM S statistic *(P-value)*	0.0000	0.0000	0.0000	0.0000
Obs.	9,826	9,826	9,826	9,826
Firms	768	768	768	768
**Panel B: Endogeneity tests for the effect of OVX on ROA and CIR variables**				
*First stage*				
EPU_US	0.4442***		0.4442***	
	(0.0021)		(0.0021)	
EPU_RUS		0.0813***		0.0813***
		(0.0023)		(0.0023)
*Second stage*				
OVX	-0.0141***	-0.1477***	0.0224***	0.1442***
	(0.0043)	(0.0187)	(0.0069)	(0.0261)
LEV	-0.0664***	-0.0633***	0.1002***	0.0974***
	(0.0111)	(0.0123)	(0.0136)	(0.0146)
SIZE	-0.0006	0.0010	-0.0152***	-0.0167***
	(0.0009)	(0.0011)	(0.0019)	(0.0020)
TANG	-0.0333***	-0.0380***	0.0483***	0.0526***
	(0.0080)	(0.0098)	(0.0163)	(0.0170)
GROWTH	0.0041***	0.0047***	-0.0059***	-0.0065***
	(0.0008)	(0.0009)	(0.0011)	(0.0011)
LIQ	0.0639***	0.0901***	-0.0533***	-0.0772***
	(0.0073)	(0.0095)	(0.0130)	(0.0147)
CF	0.1556***	0.1793***	-0.2018***	-0.2234***
	(0.0151)	(0.0174)	(0.0202)	(0.0223)
Weak-instrument-robust inference:				
Anderson-Rubin Wald test *(P-value)*	0.0000	0.0000	0.0000	0.0000
Stock-Wright LM S statistic *(P-value)*	0.0000	0.0000	0.0000	0.0000
Obs.	9,826	9,826	9,826	9,826
Firms	768	768	768	768

Note: This table reports empirical results about the impact of VOPSD on the ROA and CIR variables by using 2SLS regression, in which, EPU_US and EPU_RUS are instrumental variables. EPU_US is an average of the monthly US EPU index for the year (t). EPU_RUS is an average of the monthly Russian EPU index for the year (t). Robust standard errors clustered at the firm-level are reported in the parenthesis. *, **, and *** represent 10%, 5%, and 1% levels of significance, respectively.

Note: This table reports empirical results about the impact of OVX on the ROA and CIR variables by using 2SLS regression, in which, EPU_US and EPU_RUS are instrumental variables. EPU_US is an average of the monthly US EPU index for the year (t). EPU_RUS is an average of the monthly Russian EPU index for the year (t). Robust standard errors clustered at the firm-level are reported in the parenthesis.

*, **, and *** represent 10%, 5%, and 1% levels of significance, respectively.

## 5. Conclusions and policy implications

This paper examines how changes in oil prices affected the profitability of Chinese listed companies covering 2007 and 2020, paying particular attention to the context of China’s reform of the 2013 oil pricing regime. Empirical evidence shows that increased operating costs caused by oil price instability erode company profitability. Furthermore, detailed findings indicate that the negative effects of oil price volatility on Chinese company profitability and the positive impacts of oil price uncertainty on operating costs are more evident since the Chinese government established the 2013 oil pricing mechanisms in effect. In addition, this study proves that the detrimental effect of oil price instability on the profitability of Chinese-listed enterprises with greater market value is less apparent. Our findings implicate that government regulation and subsidies are necessary to stabilize Chinese corporate profits in the context of increasing macro risks owing to surged oil price instability.

This research supplements the corporate performance literature in the following ways. Firstly, we provide empirical evidence on the interlink between oil price volatility, profitability, and operating costs of listed companies in China, the largest oil consumer in the world. Secondly, the profitability of Chinese-listed companies is severely impacted by the instability of oil prices when the Chinese government enacts the oil pricing market-oriented regulations. Thirdly, instead of relying on retained earnings, which tend to lower due to heightened oil price volatility, large-cap firms could more easily increase external financing by issuing shares compared to small-cap enterprises. Hereby, they cope with the detrimental effect of oil price instability. This is an outstanding demonstration of the way the market capitalization scale is beneficial.

The study provides some policy implications for policymakers and corporate managers in the world’s largest import market. Firstly, our findings confirm that uncertain oil prices could strongly affect operating costs and cause adverse effects that affect the operational activities of Chinese enterprises. Therefore, policymakers should pay more attention to policies to stabilize oil prices and support Chinese enterprises. Secondly, Chinese corporate managers should have a deep perception of the adverse effect of oil price volatility on their business operations to diversify proactively fuels using the input to avoid heavy dependence on crude oil prices. Comprehending the impact of the 2013 reform’s modifications on company profitability is important, as the found effects offer justification for modifying the oil pricing mechanism subsequently. Due to limitations in accessing the database, our study has two main shortcomings. One is that the disparity in the unstable effect of oil prices on corporate profitability between state-shareholding firms and private enterprises has not been explored. Secondly, ignore the influence of the COVID-19 pandemic and the Russia-Ukrainian fray on the link between uncertain oil prices and firm performance.

## Supporting information

S1 Data (ZIP)
